# Extracellular Enzymes and Bioactive Compounds from Antarctic Terrestrial Fungi for Bioprospecting

**DOI:** 10.3390/ijerph17186459

**Published:** 2020-09-04

**Authors:** Laura Zucconi, Fabiana Canini, Marta Elisabetta Temporiti, Solveig Tosi

**Affiliations:** 1 Department of Ecological and Biological Sciences, University of Tuscia, Largo dell’Università snc, 01100 Viterbo, Italy; 2 Department of Earth and Environmental Sciences, University of Pavia, via S. Epifanio 14, 27100 Pavia, Italy; martaelisabett.temporiti01@universitadipavia.it (M.E.T.); solveig.tosi@unipv.it (S.T.)

**Keywords:** adaptative strategies, bioprospecting, extreme environment, soil fungi

## Abstract

Antarctica, one of the harshest environments in the world, has been successfully colonized by extremophilic, psychrophilic, and psychrotolerant microorganisms, facing a range of extreme conditions. Fungi are the most diverse taxon in the Antarctic ecosystems, including soils. Genetic adaptation to this environment results in the synthesis of a range of metabolites, with different functional roles in relation to the biotic and abiotic environmental factors, some of which with new biological properties of potential biotechnological interest. An overview on the production of cold-adapted enzymes and other bioactive secondary metabolites from filamentous fungi and yeasts isolated from Antarctic soils is here provided and considerations on their ecological significance are reported. A great number of researches have been carried out to date, based on cultural approaches. More recently, metagenomics approaches are expected to increase our knowledge on metabolic potential of these organisms, leading to the characterization of unculturable taxa. The search on fungi in Antarctica deserves to be improved, since it may represent a useful strategy for finding new metabolic pathways and, consequently, new bioactive compounds.

## 1. Introduction

Antarctica is characterized by the coldest and driest climate known on Earth. Within this environment, the Antarctic Peninsula and the adjacent islands (maritime Antarctica) have a cold maritime climate, with mean monthly temperatures from 0 to less than −15 °C, and annual precipitations from 350 to over 500 mm. More extreme conditions characterize the rest of the continent (continental Antarctica), with milder conditions along the coasts, becoming more extreme moving towards inland sites, up to mean monthly temperatures from −15 to −50 °C and nearly non-existent precipitation on the Ice Plateau [1]. These differences result in a greater biodiversity characterizing the maritime Antarctica and the coastal sites of continental Antarctica than the inner locations. However, in all these Antarctic regions the terrestrial ecosystems are dominated, more than those of other continents, by microorganisms, characterized by astonishing adaptations to withstand the extreme conditions of the environment [2,3]. In particular, they have to cope with extremely low temperatures, wide temperature fluctuations, frequent freeze and thaw events during the austral summer, strong winds, intense solar and UV irradiations, different pH conditions, desiccation, and locally high salinity. Microorganisms spread on the limited different substrates available, such as soils, rocks, bird feathers and dung, bryophytes, and lichens, that are sheltered niches, characterized by more buffered conditions [3,4,5].

Among microbes inhabiting soil ecosystems, fungi are ubiquitous and mainly responsible for the breaking down of organic matter and the releasing of phosphorus, nitrogen and carbon into the atmosphere and soil. They also produce and secrete a wide variety of enzymes and bioactive secondary metabolites, some of which of great biotechnological interest. Some fungal species found in the Antarctic environment are possibly present as air transported propagules from outside the continent and do not actively grow in this natural environment. Other species are indigenous, or even endemic, able to actively grow and reproduce in environmental conditions that are accounted among the most extreme on Earth [4,6,7]. These latter organisms have mostly attracted the interest of the scientific community as, to survive to this combination of environmental constraints, had to have developed multiple mechanisms of stress tolerance. These mechanisms include the activation of peculiar metabolic pathways and the production of either enzymes active at temperatures below the common limits, or other bioactive compounds of great potential value for biotechnological applications [7]. In this optic, Antarctica represents a very attractive location to search for novel cold-adapted enzymes or bioactive compounds both for being a permanently cold environment and because of the minimum human-associated activity.

Recent molecular and phylogenetic studies carried out on Antarctic and Arctic soils, sea ice cores, and cyanobacterial mats suggest that microbial diversity in harsh cold habitats may be much greater than ever appreciated [8,9]. However, even if culture-independent molecular methods, such as DNA metabarcoding, have revolutionized our understanding of microbial ecology, making possible to define the actual microbial communities composition and functionality, the traditional cultural approaches make possible to obtain isolates to be tested and exploited for their metabolic capabilities. Therefore, given the biotechnological potential of microbial enzymes or other bioactive compounds, there is a need to increase efforts for greater species recovery, as suggested for bacteria [10]. Different bioactivities have been observed among isolates of the same species, as well as different secondary metabolites produced by conspecific isolates. Considering that, it is mandatory the preservation of isolated strains in culture collections, to have available wide and unique sources of new bioactive producers [11]. New interesting results may be gained using metagenomics approaches, both to identify functional traits that drive microbial survival and community assembly, and to identify genes involved in metabolic pathways of biotechnological interest. Anyway, as stated by Duarte et al. [12], metagenomics allows one to explore new environments for putative functions, but there is still a lack of information related to the prospecting of Antarctic enzymes based on the metagenomic approach. Among the few studies published to date, the review of de Pascale et al. [13] must be taken into account, where some enzymes such as esterase, lipase, β-galactosidase, α-amilase and chitinase from polar bacteria were reported and discussed in the light of metagenomics screening. The metabolic competences of microbial communities inhabiting soil and rock surface niches in McKelvey Valley (McMurdo Dry Valleys) were also identified through GeoChip functional gene arrays and related to environmental stressors, offering tangible clues of the mechanisms behind the endurance of microorganisms in this inhospitable region [14]. Soils, in particular, comprised a unique reservoir of genes of particular applicative interest, such as those involved in transformation of organic hydrocarbons and in the lignin-like degradative pathways. Antibiotic resistance genes were also identified, indirectly indicating a level of competitive interactions between taxa, confirming data previously reported by shotgun metagenomics sequencing of these communities [15].

Despite the apparent simplicity or homogeneity of Antarctic terrestrial environments, microbial populations are often geographically structured into genetically distinct lineages. Fungi in pristine soils are more diverse than previously thought and their richness, abundance and composition are usually found to be mainly determined by environmental conditions, among which soil parameters seem to be the most prominent influencing factors [5,16,17]. In studies on Antarctic soils, the predominance of filamentous Ascomycota has been frequently reported, followed by Basidiomycota, mostly represented by yeasts; the other phyla (as Mucoromycota, Chytridiomycota, Mortierellomycota, and others) are less diverse and occasionally recorded [3,4,5,11,17].

The fungal component of continental Antarctic soils is mostly represented by few psychrophilic and many mesophilic-psychrotolerant and oligotrophic species. Among the physiological and morphological adaptations adopted by these microorganisms, the alteration of membrane lipid composition, the synthesis of different compatible solutes with protection roles of enzyme activity and with cryoprotective ability under complete dehydration (anhydrobiosis), the production of exopolysaccharides, and a strong melanin pigmentation are the most common [3].

**Figure 1:**
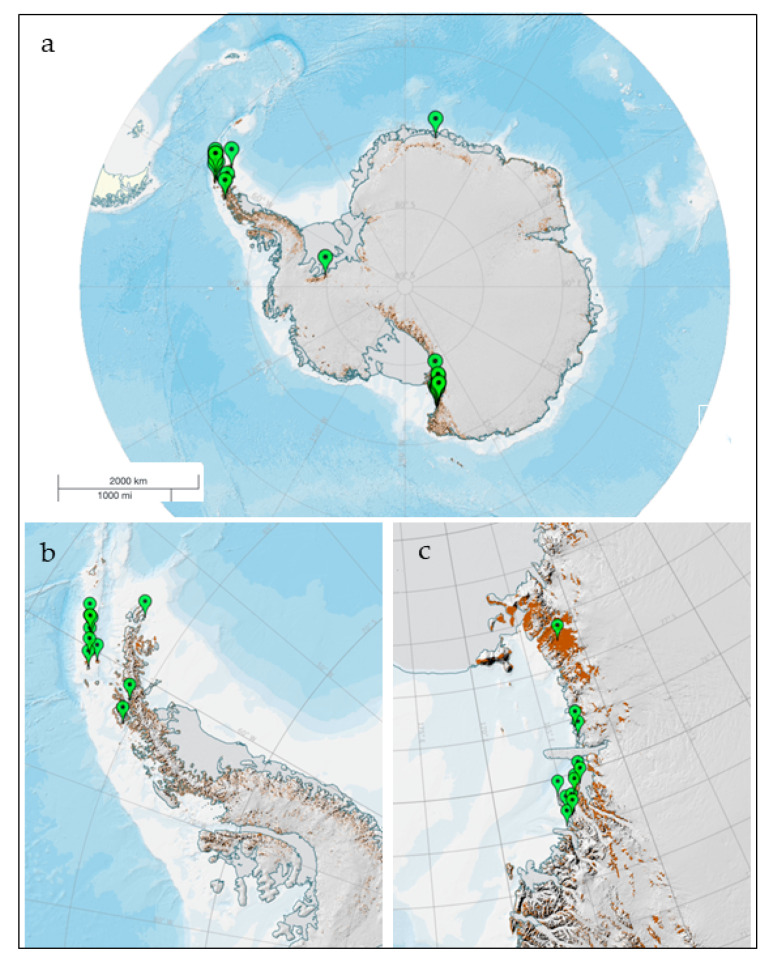
Map of the sites from where soil fungi have been isolated, producing enzymes and bioactive compounds (**a**); more detailed views of the most investigated areas, in maritime (**b**) and continental (**c**) Antarctica. Figure has been realized by the SCAR (Scientific Committee on Antarctic Research) Antarctic Digital Database (https://www.add.scar.org/).

Many studies have dealt with enzymes from cold-adapted Antarctic fungi and their potential applications, and many other research groups have reported inventory studies on species and strains able to produce bioactive prototype compounds. Given the large number of papers published, a probably not exhaustive overview is here provided; we restricted our interest to soil fungi and aimed to focus and exploit on the ecological significance of these compounds. A map of the collection sites is reported in Figure 1, from which it appears as Victoria Land in continental Antarctica and the Antarctic Peninsula and the South Shetland Islands in maritime Antarctica are the areas where the soil samples have been mostly collected, to search for enzymes and other compounds. 

## 2. Enzymes

Cold-adapted enzymes produced by psychrophilic or psychrotolerant microorganisms are an important element for the survival strategy in Antarctic ecosystems. In fact, if extremely low temperatures generally restrict microbial enzyme activity, cold-active enzymes can conduct transformations at lower temperatures than those produced by their mesophilic homologues. They display a high specific activity at low and moderate temperatures, associated with a relatively high thermosensitivity [18]. These properties make them a potentially valuable alternative to their mesophilic counterparts in cold environments. They may represent an interesting advantage in large scale processes, that generally occur at higher temperatures, for reducing the energy costs associated with heating steps [19,20,21]. While the thermosensitivity provides the possibility of rapidly inactivating them by mild heat treatments, preserving in this way the product quality [18]. Some hypotheses have been proposed to explain the cold adaptation of these enzymes compared to their mesophilic and/or thermophilic counterparts, and amino acid substitutions was supposed as a possible mechanism. These properties with regard to their current and possible applications in biotechnology have been reviewed by Marx et al. [18]. Additionally, Antarctic enzymes often exhibit a wide range of pH and temperature optima [12]. This latter property is strictly linked to wide, frequent and sudden temperature variations experienced by terrestrial surfaces, based on soil expositions and weather conditions. These wide ranges force these microorganisms to adapt to different ecological niches. A list of the enzymes produced by Antarctic soil fungi are reported in Table 1 grouped for their biological activity. The strains for which the activities have been reported, with their isolation locality, and the temperature at which the enzymatic production has been tested are reported. 

One of the widest screening for enzyme production by Antarctic fungi dates to 1997 when 33 fungal strains, belonging to 11 genera isolated from soil and mosses samples collected in different sites of Northern Victoria Land (continental Antarctica) were tested for their ability to produce a set of extracellular enzymes. The enzymes tested were among those related to the decomposition of common organic substrata: lipases, polygalacturonase, pectinlyase, amylase, cellulase, chitinase, phosphatase, glucose oxidase, urease, protease, and RNase [22]. Among these enzymes, lipases were generally produced in high quantities by almost all the strains; polygalacturonase, amylase and phosphatase were common, and glucose oxidase, protease and DNase were generally scarce or absent [22]. In particular, the psychrotolerant strain *Lecanicillium muscarium* (CCFEE 5003), formerly identified as *Verticillium lecanii*, resulted to be a powerful producer of cold-tolerant extracellular enzymes, among which chitin-hydrolyzing enzymes seemed to be the most abundant [22]. The chitinolytic activity was induced by the presence of chitin and other polysaccharides and was subjected to catabolite repression. The chitinolytic system of *L. muscarium* consisted of a number of different proteins with various molecular weights and different biochemical characteristics. Their most significant trait was the marked cold-tolerance and efficiency as mycoparasite against fungi and oomycetes. These properties make these enzymes useful to be exploited for the reuse of chitin-rich wastes and in biocontrol of pathogens [23].These chitinolytic enzymes, probably also linked to the large amounts of chitin in ornithogenic soils due to the krill-rich diet of penguins [24], showed to be active over a broad range of temperatures [25], including very low temperatures (5 °C or below) where other microorganism fail [23]. This ability to produce chitinases active at low temperatures could be very interesting in pharmaceutical industry such as for the preparation of single-cell protein, chitooligosaccharides, and N-acetyl D glucosamine. In addition, these enzymes are involved in the isolation of protoplasts from cells with chitinized walls such as fungi and yeasts, in the biological control of pathogenic fungi and insects, and in waste treatment (chitin is the second most abundant natural polymer on the Earth) [26]. A high cellulase activity at low temperature was also recorded in a *Verticillium* strain from King George Island soil. Its potential use for the development of economic and efficient systems for the bioconversion of lignocellulosic compounds into biofuels was stressed [27]. The production of ligninolytic enzymes was reported for the first time in Antarctica for fungi isolated by soil collected in different sites in the Admiralty Bay region (King George Island, Maritime Antarctica). Each characterized by different physicochemical compositions and fungal communities [28]. The NMDS (Non-metric multidimensional scaling) analysis showed fungal communities well separated by sampling site, each characterized by distinct soil physicochemical parameters. Besides differences in fungal communities composition, no correlations were computed between the enzymatic productions and the collection sites. In the face of a total of 891 isolates, the information on their enzymatic productions are very scant, with positive results mostly obtained by fungi isolated at 15 °C from root-associated soils of both *Deschampsia antarctica* and *Colobanthus quitensis* [28]. Fungi isolated from soils of an ornithogenic site close a penguin rookery, an undisturbed vegetated area, and a human impacted site at King George Island, were compared for their diversity, thermal characteristics (at 4 and 25 °C) and extracellular hydrolase enzyme production. Both cellulase and amylase activities at 25 °C tended to be greater than that at 4 °C, and both were stronger in strains isolated from the human impacted site, possibly related to an enrichment in soil organic matter. Interestingly, a greater number of strains isolated at 4 than at 25 °C were significant cellulase producers [29].

Some Antarctic fungi are able to grow despite the very low organic matter content in the soil, thanks to their wide enzymatic pattern, that increases survival chances in unfavorable environmental conditions [22]. Enzymatic activities of dehydrogenase, β-glucosidase, acid and alkaline phosphatase and arylsulphatase were detected in soils of the Dry Valleys, both amended or not with additions of C and N in both simple and complex forms. The results indicated that mineralization of organic C, P and S compounds toke place also in unamended soils, despite the very low organic matter content. The activities of almost all the enzymes were increased by C and combined C and N additions, and were either unchanged or reduced by the addition of either N only or N with only small C amounts. No evidence of shifts in the community structure as a result of the C and N supplementation was observed [30].

The high number of yeast strains isolated in Antarctica and the wide diversity of their enzyme activities highlighted the importance of these organisms in nutrient recycling in this environment. A number of yeast strains isolated from the rhizosphere of *D. antarctica*, ornithogenic (penguin guano) and not-ornithogenic soils, and other substrates in maritime Antarctica, were tested for their ability to produce secondary metabolites [31]. Cellulolytic and esterase were the most frequent activities, and most of the isolates (60%) had extracellular enzymatic activities at 4 and 20 °C. Moreover, 41.7% of the yeast strains isolated were able to produce pigments and/or mycosporines. These observations confirmed again the Antarctic mycobiota as a rich source di metabolites, involved in their adaptation to the environment, with high potential applications. For example, esterase finds application in various fields, playing an important role in the pharmaceutical industries for synthesis of chiral drugs and being able to degrade both natural materials and industrial pollutants [32]. Cellulase and esterase are suitable for degrading both agricultural waste and toxic chemicals, making them an actual interesting tool in the new frontiers of bioremediation of recalcitrant materials such as plastics [33,34]. The presence of these enzymes could also be very interesting in food, pharmaceutical, detergent industry and in waste-water treatment. The production of enzymes with low optimum temperature by Antarctic fungi allows to exploit them in processing of thermolabile food products or in bioremediation of cold water [35,36,37].

A wide screening of soil yeasts from South Shetland archipelago and Antarctic Peninsula resulted in the isolation of strains belonging to 11 genera, among which *Cryptococcus* was the most represented. All the strains showed a wide enzymatic competence, with lipase, alkaline phosphatase and invertase activities being observed in most isolates. Among the isolates *Cryptococcus gastricus* (syn. *Goffeauzyma gastrica*), *C. victoriae* (syn. *Vishniacozyma victoriae*), *C. gilvescens* (syn. *Goffeauzyma gilvencens*), *Leucosporidium* sp., and *Rhodotorula mucilaginosa* showed toxicity against other yeasts via the secretion of a protein factor, being the first report of anti-yeast activity in *Cryptococcus* and *Rhodotorula* species [38]. 

A high number of predominately psychrotolerant yeasts isolated from soil (and water) samples collected at King George Island, as *Mrakia* and *Cryptococcus* species, *Sporidiobolus salmonicolor*, *Leuconeurospora* sp., *Dioszegia fristingensis*, and the most ubiquitous *Rhodotorula laryngis* (syn. *Cystobasidium larynges*) and *Cr. victoriae*, showed extracellular activities, as lipase, amylase and esterase, while chitinase and xylanase were less common [39].

The psychrotrophic, dimorphic yeast *Candida humicola* (syn. *Vanrija humicola*), isolated from Antarctic soil, was able to secrete an acidic protease, depending on the composition of the medium. Yeast nitrogen base medium depleted of amino acids or ammonium sulphate and supplemented with proteins gave higher enzyme activity. The purified protease had a molecular mass of 36,000 Da and was inhibited by pepstatin, iodoacetamide, and sodium dodecyl sulfate. It exhibited activity over a broad range of pH values (pH 1 to 7). During exponential growth the secretion was found to be greater at low than at higher temperatures. Despite that, the enzyme was active at temperatures ranging from 0 to 45 °C, with an optimum at 37 °C [40]. This is of particular interest for the utilization of this enzyme, which may be produced at low temperatures and then used in combination with other enzymes extracted from mesophilic organisms.

A cold-adapted lipase was purified from the Antarctic filamentous fungus *Geomyces* sp. P7, isolated from soils collected in the neighborhood of the Arctowski Polish Antarctic Station at King George Island (Southern Shetlands). Its coding sequence was identified, cloned, and showed to be heterologously expressed in *Saccharomyces cerevisiae* BJ5465, retaining the same cold-adaption and thermostability characteristics of the native protein [41]. The most prominent producer of two different lipases, lipase A (CALA) and B (CALB), is an Antarctic strain of *Candida antarctica* (syn. *Moesziomyces antarcticus*), an alkali-tolerant yeast not found in soils, but in the sediment of Lake Vanda. Lipase B is probably the mostly employed hydrolase in the biocatalysts field, as an alternative to catalysts commonly used, whose residues in biomedical products are highly dangerous for human body [42].

Enzymes produced by Antarctic fungi may lead to important advancements in therapeutic applications. This is the case of 30 fungal strains isolated from soils (and mosses) of the Schirmacher Hills region (Dronning Maud Land, East Antarctica) identified as *Aspergillus* sp. IBBLA3, *Coprinopsis cinerea* IBBLA4, *Coprinopsis* sp. IBBLA5, *Trichosporon asahii* IBBLA1 and *Aspergillus niger* IBBLA2. They demonstrated to produce L-Asparaginase free of glutaminase and urease, proven to be competent in treating acute lymphoblastic leukaemia which is most common type of childhood cancer. Among the strains screened, *T. asahii* IBBLA1 exhibited the highest enzyme activity [43].

Antarctic fungi from soils of Livingstone Island in Maritime Antarctica demonstrated to be good producers of antioxidant enzymes, such as superoxide dismutase (SOD) and catalase (CAT), to survive oxidative stress, with potential application in medical and cosmetic industries. The statistically significant higher activity at 15 than at 30 °C of identified SOD suggested that this cold-active enzyme likely helps fungi in maintaining oxidant–antioxidant balance at low temperature by scavenging superoxide radicals [44].

Some strains have demonstrated promising applications in bioremediation, including the breakdown of petroleum hydrocarbons. Petroleum contamination causes extensive damage in both Arctic and Antarctica, as ecosystem recovery is slower in cold than in warm regions. In 2001, between 1 and 10 million cubic meters of soil were considered to be contaminated in Antarctica [45]. The resistance of both filamentous fungi and yeasts from soils of Adelaide Island, Antarctic Peninsula, to aromatic and aliphatic hydrocarbons was demonstrated; this research lead to the hypothesis that *Mortierella* species may be able to use dodecane as carbon and energy source [46]. Subsequently, also a number of yeasts and filamentous fungi isolated from Macquarie Island contaminated soils showed to be able to degrade hydrocarbons [10]. Finally, a *Penicillium* CHY-2 strain, isolated from an unspecified Antarctic soil produced a manganese peroxidase (MnP) degrading eight different aliphatic and aromatic hydrocarbons at low temperature conditions. This MnP consisted of monomers with a molecular mass of 36 kDa. The purified MnP had an optimum pH of 5.0 and temperature of 30 °C. The Km and Vmax values of MnP towards Mn^2+^ were 1.31 µM and 185.19 µM min^−1^ respectively. These results indicated the strain CHY-2 as possible candidate to remove hydrocarbons in contaminated polar soils [47].

A powerful tool to analyze enzymes production in fungi is represented by proteomics. Despite proteomics applications on many strains of interest in different field is increasing in the last years, the only report for Antarctic fungi is the proteomic characterization of the black meristematic fungus *Cryomyces antarcticus* that lead to the identification of many enzymatic proteins [48]. The characterization of molecular competences of this organism and possibly of other related taxa is of great interest due to its extreme resistance to environmental stresses, proved also in the exposition to ionizing radiations and space conditions (see for example [49,50]). 

## 3. Other Bioactive Compounds

In addition to enzymes, fungi are a rich reservoir of different classes of secondary metabolites, such as terpenoids, polyketides, alkaloids, polyacetylenes with demonstrated antiviral, antibacterial, antifungal, antitumoral, herbicidal and antiprotozoal activities. These molecules play a pivotal role in the inter- and intra-specific interactions within the soil microbial communities and provide them competitive advantages over other microorganisms [11,51]. A list of bioactive compounds retrieved from Antarctic soil fungi, grouped according to their biological activity is reported in Table 2.

Because of the recent emergence of antibiotic-resistant pathogenic microorganisms, and the connected risks for public health, the search for novel classes of active compounds in this context is worth to be developed for its strong practical importance, as it has been recognized by the World Health Organization as a threat to human health [52,53]. Extreme environments could be an excellent source of new antibiotics and, in this context, Antarctica, an almost unknown continent, is potentially of great interest. *Aspergillus* and *Penicillium* species are well-known producers of many bioactive compounds. *Penicillium* species are likely among the most abundant and widespread in different environments and substrates and many of them demonstrated to produce different bioactive compounds [54,55]. An overview of secondary metabolites with versatile antimicrobial potential was reported by Bratchkova and Ivanova [56] from Arctic and Antarctic microorganisms and their possible role in the adaptation and survival of microorganisms in the ice deserts was discussed. A noticeable antimicrobial activity was registered by a *P. nalgiovense* strain, from a soil sample close to an abandoned penguin nest at Paulete Island (Maritime Antarctica). This fungal strain was able to secrete the antifungal secondary metabolite amphotericin B; this ability was noteworthy, since it was the first time that amphotericin B had been isolated from an organism other than the bacterium *Streptomyces nodosus* [57]. Different fungal strains isolated from Maritime Antarctic sites showed a significant inhibitory effect on pathogenic bacteria, but a weaker effect as antimycotic [58]. Antiviral activities against the H1N1 and H3N2 influenza viruses were demonstrated by a soil strain of *Aspergillus ochraceopetaliformis* [59], thanks to the presence of compounds such as ochraceopone A, isoasteltoxin, and asteltoxin. 

Some strains are good producers of secondary metabolites with multiple activities. Godinho et al. [11] found multiple antiviral, antibacterial, antifungal, antitumoral, herbicidal and antiprotozoal activities in extracts of *Aspergillus sydowii*, *Penicillium allii-sativi*, *P. brevicompactum*, *P. chrysogenum* and *P. rubens* strains isolated from cold oligotrophic soils of the Union Glacier region in continental Antarctica. Particularly interesting were the findings of a broad high antiviral activity against Dengue virus 2, and antiprotozoal and antifungal activities by a strain of *P. brevicompactum*. The authors also observed that the same species may have different bioactivities and suggested that the low fungal diversity of the Antarctic oligotrophic soils may present a high intra-specific diversity [11]. Thus, as already stated above, it is mandatory to keep different isolates of the same species of fungi in the culture collections, to make them available for further studies.

*Penicillium tardochrysogenum* isolated from the McMurdo Dry Valleys was reported as a new endemic species of Antarctica, able to produce asperentins, penicillins, secalonic acids, and the partial characterised extrolite met Ø [60]. 

In maritime Antarctica, some endophytic fungi isolated from healthy specimens of *D. antarctica* and *C. quitensis*, and others from the rhizosphere soil of *D. antarctica*, showed to produce bioactive molecules with antimicrobial, antiprotozoal and antitumoral activities [61,62]. Extracts of 19 out of 564 of the endophytic isolates showed leishmanicidal activity and 6 inhibited the growth of at least one tumor cell line [61]. Among the root inhabitants, the extract of *Purpureocillium lilacinum* exhibited high trypanocidal, antifungal, and antibacterial activities, with moderate toxicity for normal human cells [62]. Cytotoxic metabolites with biological activity against various cancer lines were also isolated from the psychrophilic fungus *Oidiodendron truncatum* GW3-13 [63] and numerous psychrotrophic fungi isolated from different substrates collected on the Fildes Peninsula (King George Island) [64]. These latter also showed antimicrobial activity, inhibiting the growth of different bacteria in vitro, with differences among isolates of the same species [65].

New molecules with potential interesting activities, produced by Antarctic fungi, were isolated and characterized. Indeed, one new alkaloid (acremolin C) and four known compounds with weak to moderate antibacterial activities were isolated from cultures of the soil fungus *Aspergillus sydowii* [66]. New metabolites (asterric acid derivatives) with antifungal activity against *Aspergillus fumigatus* and antimicrobial activities against Gram-positive and Gram-negative bacteria were extracted from cultures of a *Geomyces* sp. strain isolated from a soil sample collected at the Fildes Peninsula, King George Island [67]. Three new metabolites were obtained from an Antarctic soil-derived *Penicillium* sp., showing cytotoxicity against the human cell lines tested and antituberculosis activity [68].

Strains of *G. pannorum*, isolated from Maritime Antarctica, gave a stronger antioxidant response, evaluated on phenolic compounds production, after cold shock [44,69]. This was suggested as a mechanism to overcome the life-endangering influence of low temperature and survive intracellular cold-induced oxidative stress. 

Great is the interest for the production of exopolysaccharides because their different applications in food, pharmaceutical, cosmetic, and other industries. Five psychrophilic exopolysaccharide producing yeast strains were isolated from Livingston Island: *Cryptococcus laurentii* (syn. *Papiliotrema laurentii*) AL65, *Sporobolomyces salmonicolor* AL36, *Debaryomyces hansenii*, *Leucosporidium scottii* and *Rhodotorula glutinis*. The highest exopolysaccharide yield was established for *L. scottii*, and significant differences in the metabolites profiling were observed using NMR spectroscopy, probably representing different adaptation strategies to Antarctic conditions, according to the phylogenetic position. Differences were observed in the production of some biotechnologically important metabolites like alanine, leucine and/or tyrosine, used for medical treatments. Some of these, along with extracellular polysaccharides, could be used in the practice as part of waste-free and more cost-effective technologies [70].

*Cryptococcus* is one of the most frequent yeast genera in this environment, with numerous new species described for the science, as *Cr. vishniacii* (syn. *Naganishia vishniacii*), *Cr. friedmannii* (syn. *Naganishia friedmannii*), *Cr. antarcticus* (syn. *Naganishia antarctica*), *Cr. albidosimilis* (syn. *Naganishia albidosimilis*) [71,72,73]. An isolate of *Cr. laurentii* (syn. *Papiliotrema laurentii*) from Antarctic soil, identified basing on its morphological, cultural and physiological properties, was selected as an active producer of exopolysaccharides, which resulted to be optimal emulsifiers with applications in cosmetic products [74].

Ocampo-Friedmann and Friedmann [75] proved a weak ability of nearly 40 cryptoendolithic fungal strains to produce biologically active substances inhibiting the growth of algae and cyanobacteria within the lichen-dominated cryptoendolithic communities of the McMurdo Dry Valleys. These substances were suggested to have a role in maintaining the zonation of microorganisms in the community. 

The search for new forms of defense from plant diseases, less harmful to the environment, has nowadays become increasingly required. Thus, many works have dealt with yeasts and filamentous fungi from Antarctic soils to assess their bioactivity potential against specific or generic pathogens. Most of the fungi tested for this purpose have been isolated from maritime Antarctic soils. A number of filamentous fungi belonging to the genera *Pseudogymnoascus* and *Cladosporium* from Deception Island (South Shetland Islands, Antarctica) soils demonstrated to produce compounds against phytopathogens such as *Xanthomonas* species [76,77]. Bioactive metabolites against neglected tropical diseases and pests were reported also from the Antarctic Peninsula: *Pseudogymnoascus destructans*, *Mortierella parvispora*, and *Penicillium chrysogenum* displayed antiparasitic activities, whilst extracts of *P. destructans*, *M. amoeboidea*, *Mortierella* sp. 3, and *P. tardochrysogenum* showed herbicidal activities [78]. The production of promising biotechnological molecules by *Mortierella* species was not a novelty, as already reported for an endophytic *M. alpina* strain isolated from *Schistidium antarctici*, a moss species widely distributed at King George Island [79].

Among Antarctic microorganisms, yeasts have been mainly suggested as the best tool for promoting the control of several phytopathologies in agriculture, for fruits storage and/or transport in cold chambers, as an important alternative to synthetic chemical fungicides. Despite that, the work in this field is still limited [80]. An inhibitory effect on the growth or germination of pathogens may be due to metabolites generated by yeasts or the production of lytic enzymes. For example, a psychrotrophic isolate of *Leucosporidium scottii* from Antarctic soils, producing soluble and volatile antifungal substances inhibiting the growth of pathogens on apples, was identified as a good biocontrol agent [81]. Moreover, two isolates of *Candida sake*, from soil and water samples collected in King Gorge Island, were reported as biocontrol agents in apples stored at low temperature, as producer of antifungal volatile organic compounds (VOCs) which inhibited the growth of five apple pathogens (*P. expansum*, *Botrytis cinerea*, *A. alternata*, *A. tenuissima*, and *A. arborescens*) [82].

Finally, some Antarctic fungal taxa exhibited a considerable melanins production. The characterization of these pigments is of great interest, due to their scavenging, radio- and photo-protective, antimicrobial, cytotoxic, anti-inflammatory, and immunomodulatory activities. As an example, their study in the Antarctic black fungus *Cryomyces antarcticus* [83] led to very interesting results in the frame of the biotechnological applications [84].

## 4. Conclusions

In 1991, a landmark paper estimated that there are 1.5 million fungi on the Earth [86]. Molecular methods have dramatically increased our knowledge on this kingdom. Subsequent estimations suggested that as many as 5.1 million fungal species exist, of which only a small percentage was known (about 10%), while the majority remained to be discovered [87]. Based on more recently generated data from culture-dependent and -independent surveys, the fungal species on the Earth have been estimated to be 12 (11.7–13.2) million at least, and an even higher diversity than this estimation is expected [88]. Therefore, fungi are an unmapped and untapped source of enzymes and secondary metabolites with an incredible application potential. Unknown fungal species and their constituent metabolites can be identified by investigating the microbiota of soil or biological samples from extreme or unexplored environments. The undiscovered microbial diversity of cold environments is today threatened by climate change. The risk of dissemination of non-indigenous microorganisms into indigenous microbial communities as consequence of climate change has been forecasted. This could result in changes of the composition of soil communities and even the disappearance of species of potential interest. Moreover, climate change that has been affecting the maritime Antarctic region for a longer time respect to the rest of the continent, concerns a real risk of dispersal of potentially pathogenic species by wild birds, which can fly great distances towards South America and Oceania. This is the case reported for some fungi potentially pathogenic to humans, isolated from ornithogenic soils of the Antarctic Peninsula, where they possibly live in a latent state [89]. 

It is common knowledge that extreme environmental conditions in Antarctica exerted an important selective pressure for many species of photosynthetic organisms, fauna and microorganisms to evolve unique characteristics and capabilities which could be used for biotechnological development. The growing commercial interest in Antarctic research raises key policy, ethical and moral questions, addressed in a report by Lohan and Johnston [90], with the inputs of many researchers, where the absence of clear rules governing the use of genetic resources from Antarctica was clearly reported. Although the products or processes based on Antarctic organisms have not been yet commercialized, bioprospecting has been deeply and carefully debated within the international research community and the Antarctic Treaty Consultative Meeting (ATCM). Despite years of discussion, there has been little progress on using the Antarctic Treaty System to regulate bioprospecting activities. As a consequence, the only resolution adopted was that of the 28th ATCM held in 2005 at Stockholm, Sweden, calling on the Treaty Parties to remind their national Antarctic programs and other research institutes engaged in Antarctic biological prospecting activities of the provisions of Article III.1 of the Treaty, concerning scientific exchanges and the availability of scientific observations and results from Antarctica (the Secretariat of the Antarctic Treaty, https://www.ats.aq/e/secretariat.html).

## Figures and Tables

**Table 1:** List of enzymes produced by Antarctic soil fungi, with their isolation site and the temperature at which the activity has been tested. Abbreviations: PG1, polygalacturonase; PG2, polypectate; PEC, pectinase; AM, amylase; CEL, cellulase; CHI, chitinase; PHO, phosphatase; GOD, glucose oxidase; URE, urease; PRO, protease; LIP, lipases; RNA, RNAase; DNA, DNAase; EST, esterase; AP, alkalin-phosphatase; INV, invertase; GEL, gelatinase; XYL, xylanase; ASP, asparaginase; SOD, superoxide dismutase; CAT, catalase; MP, manganese peroxidase; KER, keratinase.

Enzyme Category	Enzyme	Fungal Taxa	Collection Site	Temperature
Carbohydrate metabolism	PG1	*Akanthomyces lecanii* (sub. *Verticillium lecanii* sp. 2) [22]	Mt Melbourne	25 °C
		*Aspergillus versicolor* [22]	Lamplugh Island	
		*Chaetomium* sp. [22]	Mt Melbourne	
		*Cladosporium cladosporioides* [22]	Crater Cirque	
		*Dendryphiella salina* [22]	Lake “Carezza”, Baker Rocks	
		*Phoma sorghina* [22]	Kay Island	
		*Phoma* sp. [22]	Lake “Carezza”, Gondwana Station, Crater Cirque	
		*Pseudogymnoascus pannorum* (sub. *Geomyces pannorum*) [22]	Kay Island, Lake “Carezza”	
	PG2	*Alternaria* sp. [22]	Cape Washington	25 °C
		*Chaetomium* sp. [22]	Mt Melbourne	
		*Cladosporium cladosporioides* [22]	Crater Cirque	
		*C. herbarum* [22]	Crater Cirque, Whitmer Peninsula	
		*Phoma sorghina* [22]	Kay Island	
		*Pseudogymnoascus pannorum* (sub. *Geomyces pannorum*) [22]	Lake “Carezza”	
	PEC	*Alternaria* sp. [22]	Cape Washington	25 °C
		*Aspergillus versicolor* [22]	Lamplugh Island	
		*Chaetomium* sp. [22]	Mt Melbourne	25, 35 °C
		*Cladosporium herbarum* [22]	Crater Cirque, Whitmer Peninsula	25 °C
		*Cryptococcus* sp. [38]	Deception Island	10 °C
		*Cystobasidium laryngis* (sub. *Rhodotorula laryngis*) [39]	King George Island	30 °C
		*Dioszegia fristingensis* [39]		22 °C
		*Dioszegia* sp. [39]		15 °C
		*Fellozyma* sp. [38]	Snow Island	22 °C
		*Goffeauzyma gilvescens* (sub. *Cryptococcus gilvescens*) [38]	Deception Island	10 °C
		*Kriegeria* sp. [38]	Litchfield Island	30 °C
		*Leuconeurospora* sp. [31,39]	King George Island	20 °C [31]; 15° C [39]
		*Leucosporidium creatinivorum* (sub. *Leucosporidiella creatinivora*) [31]		4 °C
		*L. fragarium* (sub. *Leucosporidiella fragaria*) [39]		22 °C
		*L. muscorum* (sub. *Leucosporidiella muscorum*) [31]		4, 20 °C
		*L. scottii* [31]		4, 20 °C
		*Leucosporidium* sp. [38]	Litchfield Island	15 °C
		*Metschnikowia* sp. [39]	King George Island	10 °C
		*Mrakia frigida* (sub. *M. gelida*) [38]	Litchfield Island	10 °C
		*M. psychrophila* [39]	King George Island	10 °C
		*M. robertii* [39]		15 °C
		*Naganishia antarctica* (sub. *Cryptococcus antarcticus*) [31]		20 °C
		*Phenoliferia glacialis* (sub. *Rhodotorula glacialis*) [39]		10, 15 °C
		*Phoma sorghina* [22]	Kay Island	25 °C
		*Phoma* sp. [22]	Lake “Carezza”, Gondwana Station, Crater Cirque	
		*Pseudogymnoascus pannorum* (sub. *Geomyces pannorum*) [22]	Lake “Carezza”	
		*Sporidiobolus salmonicolor* [39]	King George Island	22 °C
	AMY	*Akanthomyces muscarius* (sub. *Lecanicillium muscarium*, det. as *Verticillium lecanii* sp. 3) [22]	Kay Island	25 °C
		*A. lecanii* (sub. *Verticillium lecanii* sp. 1, sp. 2) [22]	Crater Cirque, Mt Melbourne	
		*Alternaria* sp. [22]	Cape Washington	
		*Aspergillus versicolor* [22]	Lamplugh Island	
		*Colletotrichum* sp. (sub. *Glomerella* sp.) [29]	King George Island	25 °C
		*Cryptococcus* sp. [38]	Deception Island	10 °C
		*Dendryphiella salina* [22]	Lake “Carezza”, Baker Rocks	25 °C
		*Dioszegia fristingensis* [39]	King George Island	22 °C
		*Dioszegia* sp. [39]		15 °C
		*Fellozyma* sp. [38]	Snow Island	22 °C
		*Geomyces* spp. [29]	King George Island	4, 25 °C
		*Goffeauzyma gastrica* (sub. *Cryptococcus gastricus*) [39]		22 °C
		*G. gilvescens* (sub. *Cryptococcus gilvescens*) [39]		22 °C
		*Hamamotoa* sp. [38]	Dee Island	4–10 °C
		*Holtermanniella wattica* [39]	King George Island	30 °C
		*Hyphozyma* sp. [38]	Nelson Island	15 °C
		*Kriegeria* sp. [38]	Litchfield Island	30 °C
		*Leuconeurospora* sp. [31,39]	King George Island	4, 20 °C [31]; 15° C [39]
		*Mrakia blollopis* [39]		15 °C
		*M. frigida* (sub. *M. gelida*) [38,39]	Litchfield Island [38]; King George Island [39]	10 °C
		*M. robertii* [39]	King George Island	15 °C
		*Mrakia* sp. [39]		15 °C
		*Naganishia antarctica* (sub. *Cryptococcus antarcticus*) [31]		4, 20 °C
		*Phenoliferia glacialis* (sub. *Rhodotorula glacialis*) [39]		10, 15 °C
		*Phialemonium* sp. [29]		25 °C
		*Podospora* sp. [29]		4, 25 °C
		*Pseuderotium* sp. [29]		4 °C
		*Pseudogymnoascus pannorum* (sub. *Geomyces pannorum*) [22]	Kay Island, Lake “Carezza”, “Giardino”, “Campo Icaro”	25 °C
		*Sporothrix inflata* [29]	King George Island	25 °C
		*S. pallida* [29]		25 °C
		*S. schenckii* [29]		25 °C
		*Vishniacozyma victoriae* (sub. *Cryptococcus victoriae*) [38]	Deception Island	22 °C
		*Wardomyces inflatus* [29]	King George Island	4 °C
	CEL	*Akanthomyces lecanii* (sub. *Verticillium lecanii* sp. 1) [22]	Crater Cirque	25 °C
		*Alternaria* sp. [22]	Cape Washington	
		*Aspergillus versicolor* [22]	Lamplugh Island	
		*Candida glaebosa* [31]	King George Island	4 °C
		*C. sake* [31]		4 °C
		*Colletotrichum* sp. (sub. *Glomerella* sp.) [29]		25 °C
		*Cystobasidium* sp. [38]	Dee Island	15 °C
		*Debaryomyces hansenii* [31]	King George Island	4 °C
		*Dendryphiella salina* [22]	Lake “Carezza”	25 °C
		*Dioszegia fristingensis* [39]	King George Island	22 °C
		*Dioszegia* sp. [31]		4 °C
		*Fellozyma* sp. [38]	Snow Island	22 °C
		*Filobasidium* sp. [31]	King George Island	4, 20 °C
		*Galerina fallax* [29]		25 °C
		*Geomyces* spp. [29]		4, 25 °C
		*Goffeauzyma gastrica* (sub. *Cryptococcus gastricus*) [39]		22 °C
		*Holtermanniella wattica* [39]		30 °C
		*Hyphozyma* sp. [38]	Nelson Island	15 °C
		*Kriegeria* sp. [38]	Litchfield Island	30 °C
		*Leuconeurospora* sp. [31,39]	King George Island	4, 20 °C [31]; 15° C [39]
		*Leucosporidium creatinivorum* (sub. *Leucosporidiella creatinivora*) [31]		4, 20 °C
		*L. fragarium* (sub. *Leucosporidiella fragaria*) [31,39]		4, 20 °C [31]; 22 °C [39]
		*L. scottii* [31]		4 °C
		*Mrakia blollopis* [39]		15 °C
		*M. frigida* (sub. *M.gelida*) [38,39]	Litchfield Island [35]; King George Island [36]	10 °C
		*M. psychrophila* [39]	King George Island	10 °C
		*M. robertii* [39]		15 °C
		*Mrakia* sp. [39]		15 °C
		*Nadsonia commutata* [31]		4, 20 °C
		*Phialemonium* sp. [29]		25 °C
		*Phoma sorghina* [22]	Kay Island	25 °C
		*Phoma* sp. [22]	Lake “Carezza”, Crater Cirque	
		*Podospora* sp. [29]	King George Island	4, 25 °C
		*Pseuderotium* sp. [29]		4 °C
		*Pseudogymnoascus pannorum* (sub. *Geomyces pannorum*) [22]	Lake “Carezza”	25 °C
		*Queenslandipenidiella kurandae* (sub. *Penidiella kurandae*) [29]	King George Island	25 °C
		*Sporothrix inflata* [29]		25 °C
		*S. pallida* [29]		25 °C
		*S. schenckii* [29]		25 °C
		*Verticillium* sp. [27]	Great Wall Station, King George Island	38 °C
		*Vishniacozyma victoriae* (sub. *Cryptococcus victoriae*) [31,38,39]	King George Island [31,39]; Deception Island [38]	4, 20 °C [31]; 22 °C [38]; 15 °C [39]
		*Wardomyces inflatus* [29]	King George Island	4 °C
	INV	*Cryptococcus* sp. [38]	Deception Island	10 °C
		*Cystobasidium* sp. [38]	Dee Island	15 °C
		*Fellozyma* sp. [38]	Snow Island	22 °C
		*Goffeauzyma gilvescens* (sub. *Cryptococcus gilvescens*) [38]	Deception Island	10 °C
		*Kriegeria* sp. [38]	Litchfield Island	30 °C
		*Leucosporidium* sp. [38]		15 °C
		*Mrakia frigida* (sub. *Mrakia gelida*) [38]		10 °C
		*Rhodotorula mucilaginosa* [38]	Snow Island	30 °C
		*Sporobolomyces roseus* [38]	Deception Island	22 °C
		*Vishniacozyma victoriae* (sub. *Cryptococcus victoriae*) [38]		22 °C
	XYL	*Dioszegia fristingensis* [39]	King George Island	22 °C
	CHI	*Akanthomyces muscarius* (sub. *Lecanicillium muscarium*, det. as *Verticillium lecanii* sp. 3) [22,23]	Kay Island	25 °C
		*A. lecanii* (sub. *Verticillium lecanii* sp. 1, sp. 2) [22]	Crater Cirque, Mt Melbourne	
		*Dioszegia fristingensis* [39]	King George Island	22 °C
		*Leuconeurospora* sp. [39]		15° C
		*Metschnikowia* sp. [39]		10 °C
		*Mrakia psychrophila* [39]		10 °C
		*Pseudogymnoascus pannorum* (sub. *Geomyces pannorum*) [22]	Kay Island, Lake “Carezza”, “Giardino”, “Campo Icaro”	25 °C
		*Sporidiobolus salmonicolor* [39]	King George Island	22 °C
	GOD	*Pseudogymnoascus pannorum* (sub. *Geomyces pannorum*) [22]	Lake “Carezza”, “Campo Icaro”	25 °C
Protein metabolism	URE	*Alternaria* sp. [22]	Cape Washington	25 °C
		*Cladosporium cladosporioides* [22]	Crater Cirque	
		*Cystobasidium pallidum* [38]	Deception Island	30 °C
		*Cystobasidium* sp. [38]	Dee Island	15 °C
		*Dendryphiella salina* [22]	Lake “Carezza”	25 °C
		*Goffeauzyma gilvescens* (sub. *Cryptococcus gilvescens*) [38]	Deception Island	10 °C
		*Hamamotoa* sp. [38]	Dee Island	4, 10 °C
		*Hyphozyma* sp. [38]	Nelson Island	15 °C
		*Pseudogymnoascus pannorum* (sub. *Geomyces pannorum*) [22]	Kay Island, Lake “Carezza”, “Giardino”, “Campo Icaro”	15, 25 °C
		*Rhodotorula mucilaginosa* [38]	Snow Island	30 °C
		*Sporobolomyces roseus* [38]	Deception Island	22 °C
		*Vishniacozyma victoriae* (sub. *Cryptococcus victoriae*) [38]		22 °C
	PRO	*Akanthomyces muscarius* (sub. *Lecanicillium muscarium*, det. as *Verticillium lecan*ii sp. 3) [22]	Kay Island	20 °C
		*A. lecanii* (sub. *Verticillium lecanii* sp. 1) [22]	Crater Cirque	
		*C. herbarum* [22]	Whitmer Peninsula	
		*Cryptococcus* sp. [39]	King George Island	22 °C
		*Goffeauzyma gilvescens* (sub. *Cryptococcus gilvescens*) [39]		22 °C
		*Leuconeurospora* sp. [31,39]		4 °C [31]; 15 °C [39]
		*Leucosporidium creatinivorum* (sub. *Leucosporidiella creatinivora*) [31]		4, 20 °C
		*Leucosporidium fragarium* (sub. *Leucosporidiella fragaria*) [31]		4, 20 °C
		*Leucosporidium muscorum* (sub. *Leucosporidiella muscorum*) [31]		4, 20 °C
		*Leucosporidium scottii* [31]		4, 20 °C
		*Mrakia frigida* (sub. *M. gelida*) [39]		10 °C
		*Nadsonia commutata* [31]		4 °C
		*Sporobolomyces roseus* [38]	Deception Island	22 °C
		*Sporidiobolus salmonicolor* [39]	King George Island	22 °C
		*Vanrija humicola (sub. Candida humicola*) [40]	Schirmacher Oasis	4 °C
	GEL	*Cystobasidium laryngis* (sub. *Rhodotorula laryngis*) [38]	Litchfield Island	15 °C
		*Cystobasidium* sp. [38]	Dee Island	15 °C
		*Fellozyma* sp. [38]	Snow Island	22 °C
		*Kriegeria* sp. [38]	Litchfield Island	30 °C
		*Mrakia frigida* (sub. *M. gelida*) [38]		10 °C
		*Rhodotorula mucilaginosa* [38]	Snow Island	30 °C
		*Sporobolomyces roseus* [38]	Deception Island	22 °C
	ASP	*Aspergillus niger* [43]	Schirmacher Hills	30 °C
		*Aspergillus* sp. [43]		
		*Coprinopsis cinerea* [43]		
		*Coprinopsis* sp. [43]		
		*Trichosporon asahii* [43]		
Lipid metabolism	LIP	*Akanthomyces muscarius* (sub. *Lecanicillium muscarium*, det. as *Verticillium lecanii* sp. 3) [22]	Kay Island	25 °C
		*A. lecanii* (sub. *Verticillium lecanii* sp. 1, sp. 2) [22]	Crater Cirque, Mount Melbourne	
		*Alternaria* sp. [22]	Cape Washington	
		*Aspergillus versicolor* [22]	Lamplugh Island	
		*Chaetomium* sp. [22]	Mt Melbourne	
		*Cladosporium cladosporioides* [22]	Crater Cirque	
		*C. herbarum* [22]	Crater Cirque, Whitmer Peninsula	
		*Cryptococcus* sp. [38,39]	Deception Island [38]; King George Island [39]	10 °C
		*Cystobasidium laryngis* (sub. *Rhodotorula laryngis*) [38,39]	Litchfield Island [38]; King George Island [39]	15 °C [38]; 30 °C [39]
		*C. pallidum* [38]	Deception Island	30 °C
		*Cystobasidium* sp. [38]	Dee Island	15 °C
		*Dendryphiella salina* [22]	Lake “Carezza”, Baker Rocks	25°C, 28°C
		*Dioszegia fristingensis* [39]	King George Island	22 °C
		*Fellozyma* sp. [38]	Snow Island	22 °C
		*Geomyces* sp. [41]	King George Island	35 °C
		*Goffeauzyma gastrica* (sub. *Cryptococcus gastricus*) [39]		22 °C
		*Goffeauzyma gilvescens* (sub. *Cryptococcus gilvescens*) [38,39]	Deception Island [38]; King George Island [39]	10 °C [38]; 22 °C [39]
		*Hamamotoa* sp. [38]	Dee Island	4–10 °C
		*Hyphozyma* sp. [38]	Nelson Island	15 °C
		*Kriegeria* sp. [38]	Litchfield Island	30 °C
		*Leuconeurospora* sp. [39]	King George Island	15 °C
		*Leucosporidium creatinivorum* (sub. *Leucosporidiella creatinivora*) [31,39]		4 °C
		*Leucosporidium fragarium* (sub. *Leucosporidiella fragaria*) [39]		22 °C
		*Leucosporidium scottii* [31]		4, 20 °C
		*Leucosporidium* sp. [38]	Litchfield Island	15 °C
		*Metschnikowia* sp. [39]	King George Island	10 °C
		*Mrakia frigida* (sub. *M. gelida*) [38,39]	Litchfield Island [38]; King George Island [39]	10 °C
		*Mrakia robertii* [39]	King George Island	15 °C
		*Mrakia* sp. [39]		15 °C
		*Phenoliferia glacialis* (sub. *Rhodotorula glacialis*) [39]		10, 15 °C
		*Phoma sorghina* [22]	Kay Island	25 °C
		*Phoma* sp. [22]	Lake “Carezza”, Gondwana Station, Crater Cirque	
		*Pseudogymnoascus pannorum* (sub. *Geomyces pannorum*) [22]	Kay Island, Lake “Carezza”, “Giardino”, “Campo Icaro”	
		*Rhodotorula mucilaginosa* [38]	Snow Island	30 °C
		*Sporobolomyces roseus* [38]	Deception Island	22 °C
		*Sporidiobolus salmonicolor* [39]	King George Island	22 °C
		*Vishniacozyma victoriae* (sub. *Cryptococcus victoriae*) [31,38,39]	King Gerge Island [31,39]; Deception Island [38]	4 °C [31]; 22 °C [38]; 15 °C [39]
Nucleic acids metabolism	DNA	*Akanthomyces muscarius* (sub. *Lecanicillium muscarium*, det. as *Verticillium lecanii* sp. 3) [22]	Kay Island	25 °C
		*Chaetomium* sp. [22]	Mt Melbourne	35 °C
		*C. herbarum* [22]	Whitmer Peninsula	25 °C
		*Dendryphiella salina* [22]	Lake “Carezza”	
		*Phoma* sp. [22]	Gondwana Station, Crater Cirque	
	RNA	*Akanthomyces muscarius* (sub. *Lecanicillium muscarium*, det. as *Verticillium lecanii* sp. 3) [22]	Kay Island	25 °C
		*Aspergillus versicolor* [22]	Lamplugh Island	
		*Chaetomium* sp. [22]	Mt Melbourne	
		*Cladosporium cladosporioides* [22]	Crater Cirque	
		*C. herbarum* [22]	Crater Cirque, Whitmer Peninsula	
		*Phoma* sp. [22]	Lake “Carezza”	
		*Pseudogymnoascus pannorum* (sub. *Geomyces pannorum*) [22]	“Campo Icaro”	
Antioxidant enzymes	SOD	*Aspergillus glaucus* [44]	Livingston Island	20, 25 °C
		*Aspergillus* spp. [44]		25 °C
		*Cladosporium cladosporioides* [44]		15 °C
		*C. herbarum* [44]		20 °C
		*C. oxysporum* [44]		15 °C
		*Epicoccum nigrum* [44]		15, 20 °C
		*Monodictys austrina* [44]		20 °C
		*Penicillium aurantiogriseum* [44]		
		*P. dierckxii* [44]		
		*P. italicum* [44]		25 °C
		*P. olsonii* [44]		20 °C
		*P. waksmanii* [44]		
		*Penicillium* spp. [44]		15, 20 °C
		*Pseudogymnoascus pannorum* (sub. *Geomyces pannorum*) [44]		20, 25 °C
		*Rhizopus* sp. [44]		25 °C
	CAT	*Aspergillus glaucus* [44]	Livingston Island	20, 25 °C
		*Aspergillus* spp. [44]		25 °C
		*Cladosporium cladosporioides* [44]		15 °C
		*C. herbarum* [44]		20 °C
		*C. oxysporum* [44]		15 °C
		*Epicoccum nigrum* [44]		15, 20 °C
		*Monodictys austrina* [44]		20 °C
		*Penicillium aurantiogriseum* [44]		20 °C
		*P. dierckxii* [44]		20 °C
		*P. italicum* [44]		25 °C
		*P. olsonii* [44]		20 °C
		*P. waksmanii* [44]		
		*Penicillium* spp. [44]		15, 20 °C
		*Pseudogymnoascus pannorum* (sub. *Geomyces pannorum*) [44]		20, 25 °C
		*Rhizopus* sp. [44]		25 °C
	MP	*Penicillium* sp. [47]	Not specified Antarctic site	20 °C
Other hydrolithic enzymes	PHO	*Akanthomyces muscarius* (sub. *Lecanicillium muscarium*, det. as *Verticillium lecanii* sp.3) [22]	Kay Island	25 °C
		*A. lecanii* (sub. *Verticillium lecanii* sp. 1, sp. 2) [22]	Crater Cirque, Mount Melbourne	
		*Aspergillus versicolor* [22]	Lamplugh Island	
		*Chaetomium* sp. [22]	Mt Melbourne	
		*C. herbarum* [22]	Crater Cirque, Whitmer Peninsula	
		*Phoma sorghina* [22]	Kay Island	
		*Pseudogymnoascus pannorum* (sub. *Geomyces pannorum*) [22]	“Giardino”, “Campo Icaro”	
	EST	*Cryptococcus* sp. [38]	Deception Island	10 °C
		*Cystobasidium laryngis* (sub. *Rhodotorula laryngis*) [38,39]	Litchfield Island [38]; King George Island [39]	15 °C [38]; 30 °C [39]
		*C. pallidum* [38]	Deception Island	30 °C
		*Dioszegia crocea* [31]	King George Island	4, 20 °C
		*D. fristingensis* [39]		22 °C
		*D. hungarica* [31]		4, 20 °C
		*Dioszegia* sp. [31,39]		4, 20 °C [31]; 15 °C [39]
		*Glaciozyma antarctica* [39]		10 °C
		*Goffeauzyma gastrica* (sub. *Cryptococcus gastricus*) [39]		22 °C
		*G. gilvescens* (sub. *Cryptococcus gilvescens*) [38]	Deception Island	10 °C
		*Hyphozyma* sp. [38]	Nelson Island	15 °C
		*Leuconeurospora* sp. [31,39]	King George Island	4 °C [31]; 15 °C [39]
		*Leucosporidium creatinivorum* (sub. *Leucosporidiella creatinivora*) [31,39]		4, 20 °C [31]; 22 °C [39]
		*L. fragarium* (sub. *Leucosporidiella fragaria*) [31,39]		4, 20 °C [31]; 22 °C [39]
		*L. scottii* [31]		4, 20 °C
		*Leucosporidium* sp. [38]	Litchfield Island	15 °C
		*Mrakia blollopis* [39]	King George Island	15 °C
		*M. psychrophila* [39]		10 °C
		*Nadsonia commutata* [31]		4, 20 °C
		*Phenoliferia glacialis* (sub. *Rhodotorula glacialis*) [39]		15 °C
		*Sporobolomyces roseus* [38]	Deception Island	22 °C
		*Vishniacozyma victoriae* (sub. *Cryptococcus victoriae*) [31,38,39]	King George Island [31,39]; Deception Island [38]	4, 20 °C [31]; 22 °C [38]; 15 °C [39]
	AP	*Cryptococcus* sp. [38]	Deception Island	10 °C
		*Cystobasidium laryngis* (sub. *Rhodotorula laryngis*) [38]	Litchfield Island	15 °C
		*Cystobasidium pallidum* [38]	Deception Island	30 °C
		*Cystobasidium* sp. [38]	Dee Island	15 °C
		*Fellozyma* sp. [38]	Snow Island	22 °C
		*Goffeauzyma gilvescens* (sub. *Cryptococcus gilvescens*) [38]	Deception Island	10 °C
		*Hamamotoa* sp. [38]	Dee Island	4–10 °C
		*Kriegeria* sp. [38]	Litchfield Island	30 °C
		*Leucosporidium* sp. [38]		15 °C
		*Mrakia frigida* (sub. *M. gelida*) [38]		10 °C
		*Rhodotorula mucilaginosa* [38]	Snow Island	30 °C
		*Sporobolomyces roseus* [38]	Deception Island	22 °C
		*Vishniacozyma victoriae* (sub. *Cryptococcus victoriae*) [38]		

**Table 2:** Bioactive compounds produced by soil fungi, grouped according to their activity. Fungal taxa and target organisms or tumoral cell lines are reported. Biological activities without a detected chemical compound are reported as “not analyzed”. Compounds not expressing a biological activity are reported as “checked activity not significant”. Compounds not checked for their activity are reported as “not evaluated”. Further details about the isolation locality of each strain, the temperature at which the activities were tested and other notes are available in Table S1.

Activity	Chemical Category	Bioactive Secondary Metabolite	Fungal Taxa and Reference	Active against the Target Organism/Cell Line
Cytotoxic/antitumoral	Alkaloid	Neoxaline	*Penicillium* sp. [68]	K562, MCF-7, A549, U937, Hela, DU145, HL60, and HT29 cell lines
		Meleagrin		
		Questiomycin A		
		Chetracin	*Oidiodendron truncatum* [64]	P388 lymphocytic leukemic cell line
		Chetracin B, C (new epi-polythiodioxopiperazines)		HCT-8 (human colon cancer cell line), BEL-7402 (human hepatocellular carcinoma cell line), BGC-823 (human gastric carcinoma cell line), A-549 (human lung cancer cell line), A-2780 (human ovarian carcinoma cell line)
		Chetracin D (new diketopiperazine)		
		Melinacidin IV (epi-polythiodioxopiperazine)		
		T988 A		
		T988 C		
	Dimeric tetrahydro-xanthones (phenolic derivative)	Secalonic acid	*Cladosporium sphaerospermum* [64]	P388 lymphocytic leukemic cell line
		Not Analyzed	*Aspergillus aculeatus* [64]	P388 lymphocytic leukemic cell line
			*A. flavus* [64]	
			*A. terreus* [64]	
			*Microdochium phragmitis* [61]	human cancer cell lines MCF-7 (breast)
			*Microdochium* sp. [61]	
			*Mortierella antarctica* [64]	P388 lymphocytic leukemic cell line
			*Mortierella* sp. [64]	
			*Mycosphaerella tassiana* (sub. *Davidiella tassiana*) [61]	human cancer cell lines MCF-7 (breast)
			*Penicillium chrysogenum* [64]	P388 lymphocytic leukemic cell line
			*P. citrinum* [64]	
			*P. solitum* (sub. *P. crustosum*) [64]	
			*Pseudogymnoascus* sp. [64]	
			*Rhizoscyphus* sp. [64]	
			*Aspergillus sydowii* [11]	cancer cell lines MCF-7 (breast)
			*Penicillium allii-sativi* [11]	
			*P. brevicompactum* [11]	
Antibacterial	Alkaloid	Acremolin C (8-isopropyl-1, 5-dimethyl-1H-imidazo[2,1-b]purin-4(5H)-one)	*Aspergillus sydowii* [66]	methicillin-resistant *Staphylococcus epidermidis* (MRSE) and methicillin-resistant *Staphylococcus aureus* (MRSA), *S. epidermidis* (ATCC 040188), *S. aureus* (ATCC 25923)
		Cyclo-(L-Trp-L-Phe), cyclic dipeptide (new compound)		
		Questiomycin A	*Penicillium* sp. [68]	*Mycobacterium tuberculosis*
	Phenolic derivative	Geomycin C (new asterric acid derivative)	*Geomyces* sp. [67]	*Staphylococcus aureus* (ATCC 6538), *Streptococcus pneumoniae* (CGMCC 1.1692), *Escherichia coli* (CGMCC 1.2340)
	Sesquiter-penoid and phenolic acid	(7S)-(+)-hydroxysydonic acid	*Aspergillus sydowii* [66]	methicillin-resistant *Staphylococcus epidermidis* (MRSE) and methicillin-resistant *Staphylococcus aureus* (MRSA), *S. epidermidis* (ATCC 040188), *S. aureus* (ATCC 25923)
		(7S, 11S)-(+)-12-hydroxysydonic acid		
		Not Analyzed	Anamorphic fungi [58]	*Pseudomonas aeruginosa* (ATCC 27853), *Escherichia coli* (ATCC 25922)
			*Aspergillus fumigatus* [58]	*Bacillus subtilis* (ATCC 6051)
			*Kabatiella zeae* [58]	*Escherichia coli* (ATCC 25922), *Bacillus subtilis* (ATCC 6051), *Staphylococcus aureus*, *Pseudomonas aeruginosa* (ATCC 27853)
			*Mucor* sp. [58]	*Staphylococcus aureus*
			*Penicillium chrysogenum* [58]	*Staphylococcus aureus*, *Bacillus subtilis* (ATCC 6051)
			*Antarctomyces psychrotrophicus* [58]	*Escherichia coli* (ATCC 25922)
			*Geomyces* sp. [58]	*Escherichia coli* (ATCC 25922), *Bacillus subtilis* (ATCC 6051), *Staphylococcus aureus*
			*Aspergillus sydowii* [11]	*Staphylococcus aureus* (ATCC 12600)
			*Penicillium allii-sativi* [11]	
			*Penicillium brevicompactum* [11]	
			*P chrysogenum* [11]	
			*Oidiodendron truncatum* [64]	*Mycobacterium phlei*; *Staphylococcus aureus*
			*Aspergillus terreus* [64]	*Escherichia coli; Proteus mirabilis; Mycobacterium phlei*
			*Cladosporium sphaerospermum* [64]	*Staphylococcus aureus*
			*Penicillium oxalicum* [64]	*Escherichia coli; Mycobacterium phlei; Proteus mirabilis; Staphylococcus aureus*
			*P. solitum* (sub. *P. crustosum*) [64]	
			*Pseudogymnoascus* sp. [64]	*Escherichia coli; Mycobacterium phlei; Proteus mirabilis*
			*Purpureocillium lilacinum* [62]	*Staphylococcus aureus*
			*Pyricularia* sp. [64]	*Escherichia coli*
Antifungal	Acyclic monoterpenoid	3,7-Dimethyl-6-octen-1-ol (citronellol) one of the main constituents of the VOCs	*Candida sake* [82]	postharvest pathogens of apple during cold storage: *Penicillium expansum, Botrytis cinerea, Alternaria alternata, A. tenuissima,* and *A. arborescens*
	Aromatic compound and alcohol	phenylethyl alcohol, one of the main constituents of the VOCs		
	Fatty acid ester	3-methylbutyl hexanoate, the main constituents of VOCs		
	Phenolic derivative	Geomycin B (new asterric acid derivative)	*Geomyces* sp. [67]	*Aspergillus fumigatus* (ATCC 10894)
	Polyene macrolide	Amphotericin B	*Penicillium nalgiovense* [57]	*Candida albicans* (ATCC 90028)
		Not Analyzed	*Aspergillus sydowii* [11]	*Cladosporium sphaerospermum* (CCT 1740)
			*Penicillium allii-sativi* [11]	
			*P. brevicompactum* [11]	
			*P. chrysogenum* [11]	*Colletotrichum gloesporioides, Colletotrichum fragariae, Cladosporium sphaerospermum* (CCT 1740)
			*P. rubens* [11]	*Cladosporium sphaerospermum* (CCT 1740)
			*Aspergillus flavus* [64]	*Candida albicans*
			*A. terreus* [64]	
			*Beauveria bassiana* [62]	*Paracoccidioi-des brasiliensis*
			*Fusarium avenaceum* (sub. *Gibberella avenacea*) [62]	*Paracoccidioides brasiliensis*
			*Leucosporidium scottii* [81]	*Penicillium expansum, Botritys cinerea*
			*Oidiodendron truncatum* [64]	*Candida albicans*
			*Penicillium chrysogenum* [64]	
			*P. citrinum* [64]	
			*P. oxalicum* [64]	
			*P. solitum* (sub. *P. crustosum*) [64]	
			*Penicillium* sp. [62]	*Paracoccidioides brasiliensis*
			*Peniophora* sp. [62]	
			*Pestalotiopsis microspora* [62]	
			*Phanerochaete* sp. [62]	
			*Pseudeurotium* sp. [62]	
			*Pseudogymnoascus* sp. [62]	
			*Purpureocillium lilacinum* [62]	
			*Schizophyllum commune* [62]	
			*Simplicillium lamellicola* [62]	
			*Trichoderma longibrachiatum* [62]	
			*Trichosporon asteroides* [62]	
Anti-protozoal		Not Analyzed	*Penicillium brevicompactum* [11]	*Trypanosoma cruzi*
			*Alternaria* sp. [61]	*Leishmania amazonensis* (strain IFLA/BR/196/PH-8)
			*Antarctomyces psychrotrophicus* [61]	
			*Cadophora luteo-olivacea* [61]	
			*Helgardia* sp. [61]	
			*Herpotrichia* sp. [61]	
			*Oculimacula* sp. [61]	
			*Phaeosphaeria herpotrichoides* [61]	
			*Phaeosphaeria* sp. [61]	
			*Purpureocillium lilacinum* [62]	*Trypanosoma cruzi*
			*Mortierella amoeboidea* [78]	*Leishmania amazonensis* (strain IFLA/BR/196/PH-8)
			*M. parvispora* [78]	*Trypanosoma cruzi, Leishmania amazonensis* (strain IFLA/BR/196/PH-8)
			*Penicillium chrysogenum* [78]	*Leishmania amazonensis* (strain IFLA/BR/196/PH-8)
			*Pseudogymnoascus destructans* [78]	
Antiviral	Furofuran-triene α-pyrone	Asteltoxin	*Aspergillus ochraceopetali-formis* [59]	H1N1 and H3N2 influenza viruses
		Isoasteltoxin		
	α-Pyrone merosesqui-terpenoid	Ochraceopone A		
		Not Analyzed	*Penicillium allii-sativi* [11]	Dengue virus 2
			*P. brevicompactum* [11]	
			*P. chrysogenum* [11]	
Herbicidal		Not Analyzed	*Penicillium chrysogenum* [11]	*Lactuca sativa* (lettuce) and *Agrostis stolonifera* (bentgrass)
			*Mortierella amoeboidea* [78]	
			*Mortierella* sp. [78]	
			*Penicillium tardochrysogenum* [78]	
			*Pseudogymnoascus destructans* [78]	*Agrostis stolonifera* (bentgrass)
Checked activity not significant	Alkaloid	Chetoseminudin C	*Oidiodendron truncatum* [63]	
		(*E*)-3-(1*H*-imidazole-4-ylmethylene)-6-(1*H*-indl-3-ylmethyl)-2,5-piperazinediol	*Penicillium* sp. [68]	
		Isopenilline A (new indolyl diketopiperazine derivative)	*Penicillium* sp. [68]	
		Oidioperazine A, B, C, D (new diketopiperazines)	*Oidiodendron truncatum* [63]	
		Penilline A (new indolyl diketopiperazine derivative)	*Penicillium* sp. [68]	
		Penilline B (new indolyl diketopiperazine derivative)		
		Penilloid A (new compound)		
		cyclo-L-Trp-Lser (cyclic dipeptid)	*Oidiodendron truncatum* [63]	
		T988 B		
	Carboxylic acid and phenol	4-hydroxyphenylacetic acid	*Aspergillus sydowii* [66]	
	Phenolic derivative and ester	Ethyl asterrate (new diphenyl ether)	*Geomyces* sp. [67]	
	Phenolic derivative and ester	*n*-butyl asterrate (new diphenyl ether)		
	Phenolic derivative	asterric acid		
	Phenolic derivative and acid	Methyl asterrate		
	Phenolic derivative and acid	Geomycin A (new asterric acid derivative)		
	Phenolic derivative (spiro compound)	Bisdechloro-geodin		
	Merosesquiterpenoid	ochraceopone B, C, D, E (new highly oxygenated α-pyrone merosesquiter-penoids)	*Aspergillus ochraceopetaliformis* [59]	
Activity not evaluated	γ-Amino acid	γ-Aminobutyric acid (GABA)	*Debaryomyces hansenii* [70]	
	Beta-lactam derivatives	Penicillins	*Penicillium tardochrysogenum* [60]	
	Carboxylic acid amide	*N*-(2-hydroxyphenyl)-acetamide	*Penicillium* sp. [68]	
	Carboxylic acid	Acetic acid	*Debaryomyces hansenii* [70]	
		Formic acid		
			*Rhodotorula glutinis* [70]	
	Dimeric tetrahydro-xanthones (phenolic derivative)	Secalonic acid D, F	*Penicillium tardochrysogenum* [60]	
	Exopolysaccharides	Exopolysaccharides	*Cryptococcus laurentii* AL62 [85]	
			*Debaryomyces hansenii* [70]	
			*Rhodotorula glutinis* [70]	
	Heterocyclic compound	2-Benzoxazolinone	*Penicillium* sp. [68]	
	Isocoumarins (phenolic derivatives)	Asperentins	*P. tardochryso-genum* [60]	
	Primary alchool	Choline	*Cryptococcus laurentii* [70]	
			*Debaryomyces hansenii* [70]	
			*Leucosporidium scotii* [70]	
			*Rhodotorula glutinis* [70]	
			*Sporobolomyces salmonicolor* [70]	
		partially characterized ‘met Ø’	*Penicillium tardochrysogenum* [60]	

## Supplementary Materials

The following is available online at https://www.mdpi.com/1660-4601/17/18/6459/s1, Table S1: Bioactive compounds produced from soil fungi, grouped according to their biological activity. Names of fungal taxa, isolation sites, temperature and target organisms or tumoral cell lines are reported. Biological activities without a detected chemical compound are reported as “not analyzed”. Compounds not expressing a biological activity are reported as “checked activity not significant”. Compounds not checked for their biological activity are reported as “not evaluated”. Notes refer to strains isolation or growth temperatures.
